# The mechanism of galvanic/metal-assisted etching of silicon

**DOI:** 10.1186/1556-276X-9-432

**Published:** 2014-08-26

**Authors:** Kurt W Kolasinski

**Affiliations:** 1 Department of Chemistry, West Chester University, West Chester, PA 19383-2115, USA

**Keywords:** Nanowires, Porous silicon, Nanostructures, Reaction dynamics, Electron transfer, Stain etching, Galvanic etching, Metal-assisted etching

## Abstract

Metal-assisted etching is initiated by hole injection from an oxidant catalyzed by a metal nanoparticle or film on a Si surface. It is shown that the electronic structure of the metal/Si interface, i.e., band bending, is not conducive to diffusion of the injected hole away from the metal in the case of Ag or away from the metal/Si interface in the cases of Au, Pd, and Pt. Since holes do not diffuse away from the metals, the electric field resulting from charging of the metal after hole injection must instead be the cause of metal-assisted etching.

## Background

Electroless etching of silicon induced by an oxidant in acidic fluoride solutions was first described by Fuller and Ditzenberger [1], Turner [2], and Archer [3] in a regime that produces nanocrystalline porous silicon. These porous films exhibit colors induced by white light interference effects and scattering; hence, they were called stain films and the process stain etching. Kolasinski [4-6] has recently unambiguously demonstrated that hole injection into the Si valence band initiates etching and is the rate-determining step in the overall etch process. Furthermore, the connection of hole injection to the electronic structure of Si is what leads to the inherently self-limiting nature of stain etching that produces nanostructures. This is because quantum confinement leads to a downward shift in the valence band when Si features drop below approximately 2 nm in a critical dimension. The downward shift of the valence band with decreasing feature size decreases the rate of hole injection into the pore walls of the porous film, which effectively passivates the walls toward further electroless etching.

Two extremely versatile variations on stain etching have gained considerable interest because they are capable of producing not only patterned films within Si devices but also ordered arrays of pores or nanowires [7,8]. The first process is called galvanic etching. It was demonstrated in a controlled manner by Kelly and co-workers [9-12]. In galvanic etching, a planar metal film is deposited on a wafer (either on the front face or on the back face). Upon exposure of the wafer to an oxidant + HF solution, the metal catalyzes hole injection from the oxidant. The second process is metal-assisted etching. Li and Bohn [13] recognized that the presence of metal localizes etching to the vicinity of the metal. Therefore, if well-spaced metal nanoparticles are used as a catalyst, pores can be etched. If a metal film with an array of openings is deposited, the substrate beneath the metal is etched with the unetched Si beneath the openings being left as nanowires with roughly the same size as the openings.

The purposes of this report are to demonstrate that the mechanism proposed in the literature to explain both galvanic and metal-assisted etching is incorrect and to propose a new one on the basis of an understanding of the band structure of the system.

The mechanism proposed in the literature [7,12,13] to explain galvanic and metal-assisted etching is analogous to stain etching. In stain etching, a hole is injected directly into the Si valence band wherever the oxidant collides with the surface. Direct measurements of etch rates and comparison to Marcus theory demonstrated [5] that each hole injected is used to etch one Si atom. Because of the random nature of oxidant/surface collisions, optimized stain etching produces thin films of porous Si (por-Si) with randomized pores but uniform lateral porosity (porosity gradients from top to bottom of the film are observed for thick films). In contrast, metal-assisted etching is concentrated on the region of the metal/Si interface.

There are, however, several problems with the literature model of metal-assisted etching. First, as shown in many reports [7,8], the pore left by the etch track of a metal nanoparticle is usually surrounded by a microporous region. Within the literature model, this is ascribed to holes diffusing into the Si away from the metal. Second, if holes are produced at the metal/Si interface - which lies at the bottom of the metal nanoparticle not exposed to the solution - how is the HF solution transported there to facilitate etching? Third, why does the hole leave the metal since the Fermi level lies above the bulk Si valence band?

The transport of holes is determined by the band structure of the metal/Si interface. Hot holes injected far below *E*_F_ will relax to *E*_F_ in less than a femtosecond. At the Fermi velocity, this means that they can travel no more than a few nanometers before they cool to the top of the band. In any case, according to Marcus theory, the majority of holes are injected at *E*_F_. Thus, we need not consider hot hole transport. Below, we will show that an approximate calculation of the electronic structure at the metal/Si interface using the Schottky-Mott relationships [14,15] does not support the idea of hole diffusion away from the metal/Si interface. Instead, the charge stays on the metal nanoparticle, which generates an electric field. The charged metal then effectively acts like a localized power supply that induces anodic etching.

## Methods

Etching was performed on (1) Si(100) 0 to 100 Ω cm p-type test grade wafers, (2) Si(111) mechanical grade n-type wafers (both University Wafer, Boston, MA, USA), or (3) unpolished single crystal reclaimed wafer chunks (Union Carbide Corporation, Houston, TX, USA). Chunks were sieved to obtain a narrow size distribution (3.35 to 4.75 mm). The sample size was large enough (approximately 2 g) to ensure constant initial surface area. The silicon was cleaned by ultrasonication in acetone then ethanol followed by rinsing in water. After etching, samples were rinsed in water and ethanol, then dried in a stream of Ar gas. V_2_O_5_ (Fisher certified grade (Thermo Fisher Scientific, Waltham, MA, USA)), HOOH (EMD Chemical (Gibbstown, NJ, USA), 30% solution in water), and HF (JT Baker (Phillipsburg, NJ, USA), 49% analytical grade) were used to create stain etchants. Metal deposition was performed galvanically by adding a few drops of 0.1 to 1 mM metal salt solution to HF, resulting in metal coverage of about 5% of the Si surface. The Si wafers with metal deposits were then transferred directly to the stain etchant with a droplet of deposition solution covering the wafer. In this manner, the H-terminated surface and the deposited metal nanoparticles were never exposed to the atmosphere and potential contamination. Aqueous salt solutions used for deposition include PdCl_2_ (Sigma-Aldrich (St. Louis, MO, USA), reagent plus, 99%), AgNO_3_ (ACS certified, >99.7%), H_2_PtCl_6_ (EMD Chemical, 10% (*w*/*w*) solution), and CuCl (Allied Chemical (Morristown, NJ, USA), reagent grade 98%).

## Results and discussion

The Fermi energy of intrinsic Si, *E*_i_, lies in the middle of the band gap equidistant from the conduction band minimum *E*_C_ and the valence band maximum *E*_V_. Based on the doping level, the Fermi energy of doped Si *E*_F_ shifts up in n-type or down in p-type Si according to

(1) $$E_{F}-E_{i}=k_{B}T\ln\left(n_{D}/n_{i}\right)$$

(2) $$E_{i}-E_{F}=k_{B}T\ln\left(n_{A}/n_{i}\right),$$

where *n*_i_ is the intrinsic density of donors in Si, *n*_D_ is the donor density in n-type Si and *n*_A_ is the acceptor density in p-type Si. From the work of Novikov [16], the value of the intrinsic work function can be obtained, *E*_*i*_ = 4.78 ± 0.08 eV. The intrinsic donor density is *n*_i_ = 1.08 × 10^10^ cm^-3^ at 300 K [15]. Here, I use typical donor densities of *n*_D_ = 1 × 10^15^ cm^-3^, which corresponds to 5 Ω cm, and *n*_A_ = 1 × 10^15^ cm^-3^, which corresponds to 14 Ω cm. Accordingly, *E*_F_ - *E*_*i*_ = 0.296 eV on n-type Si and *E*_*i*_ - *E*_F_ = 0.296 eV on p-type Si. The doping density is not critical as changing the values from 10^14^ cm^-3^ to 10^16^ cm^-3^ will only change *E*_F_ - *E*_*i*_ by ±0.06 eV, i.e., less than the uncertainty in *E*_i_.

These values are used to calculate the work function of Si, *Φ*_S_ (see Table 1). The positions of the Si bands are calculated with a Schottky-Mott analysis. This analysis assumes that (i) the Fermi energy of a metal and semiconductor in electrical contact is equal throughout both materials, (ii) the vacuum energy of Si varies smoothly and is only equal to that of the metal at the interface, and (iii) the electron affinity and band gap of Si are constant. Ideal Schottky barrier heights ($E_{b,n}^{\mathrm{ideal}}$ and $E_{b,p}^{\mathrm{ideal}}$ for n-type and p-type Si, respectively) and band positions in the absence of formation of surface states and reconstruction at the interface are calculated according to the Schottky-Mott relationships [14,15,17].

**Table 1:** **Work function ***Φ***, experimental Schottky barrier on *n*-type Si**$E_{b,n}^{\text{exp}}$**, calculated Schottky barriers,**$E_{b,n}^{\mathrm{ideal}}$**and**$E_{b,p}^{\mathrm{ideal}}$**, and standard electrochemical potential** ***E°***

	***Φ*/eV**	$E_{b,n}^{\text{exp}}/\mathrm{eV}$	$E_{b,n}^{\mathrm{ideal}}/\mathrm{eV}$	$E_{b,p}^{\mathrm{ideal}}/\mathrm{eV}$	***E°*/V**
Ag	4.74	0.60 ± 0.03 [18]	0.69	0.43	0.7996
Au	5.31	0.84 ± 0.02 [19]	1.26	-0.14	1.498
Pd	5.6	0.75 [20]	1.55	-0.43	0.951
Pt	5.93	0.85 [20]	1.88	-0.76	1.18
Si	4.48 n-type	Equation 1	*χ*_S_ = 4.05	*E*_g_ = 1.12	Approximately 0.7 (*E*_V_)
	5.08 p type	Eq. (2)			

The Si work functions are calculated for a doping density of 1 × 10^15^ cm^-3^. The values of the Si electron affinity *χ*_s_ and band gap *E*_*g*_ are taken from Sze [15]. The electrochemical potential of the Si valence band is taken from [17]. Metal work functions for (111) plane and *E°* are taken from [21].

(3) $$E_{b,n}^{\text{ideal}}=\Phi_{M}-\chi_{S}$$

(4) $$E_{b,p}^{\text{ideal}}=\chi_{S}+E_{g}-\Phi_{M}$$

(5) $$E_{\text{vac}}\left(z\right)=E_{\text{vac},\;\text{Si}\;\text{bulk}}+\Phi_{D}\left(z\right)$$

(6) $$E_{C}\left(z\right)=E_{\text{vac},\;\text{Si}\;\text{bulk}}-\chi_{S}+\Phi_{D}\left(z\right)$$

(7) $$E_{V}\left(z\right)=E_{\text{vac},\;\text{Si}\;\text{bulk}}-\chi_{S}-E_{g}+\Phi_{D}\left(z\right)$$

(8) $$\Phi_{D}\left(z,=,0\right)=\Phi_{D}=\Phi_{M}-\Phi_{S}$$

**Figure 1:**
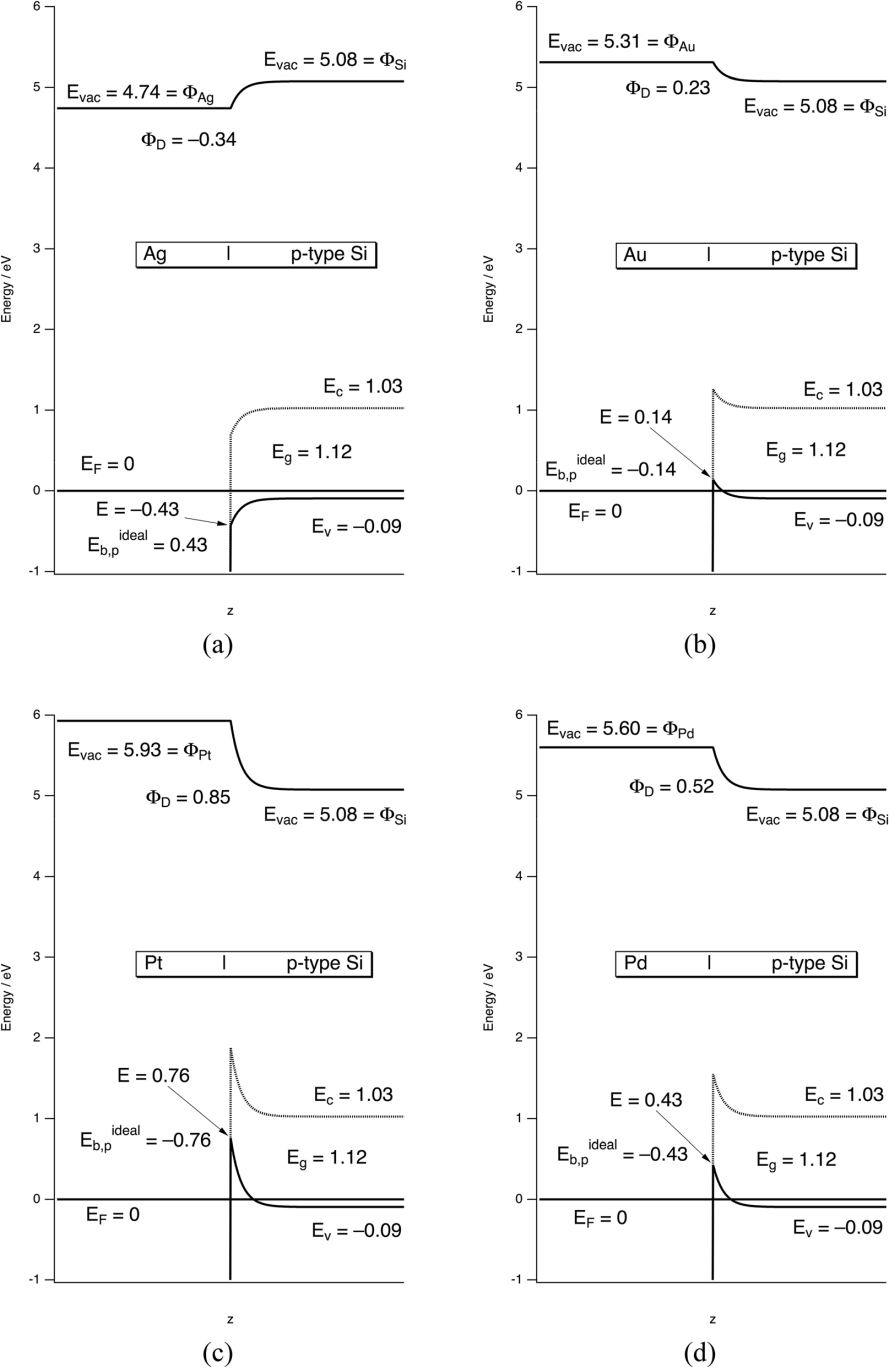
**Band bending at the metal/p-type Si interface for (a) Ag, (b) Au, (c) Pt, and (d) Pd.***E*_vac_ = the vacuum energy. *Φ*_M_ = metal work function. *Φ*_Si_ = Si work function. *E*_*g*_ = Si band gap. *E*_*F*_ = Fermi energy. *E*_*C*_ = Si conduction band energy. *E*_*V*_ = Si valence band energy. *Φ*_D_ = maximum band bending. The value *E* indicates the energy of the Si valence band directly at the metal/Si interface. $E_{b,p}^{\mathrm{ideal}}$ is the Schottky barrier height from Equation 4.

**Figure 2:**
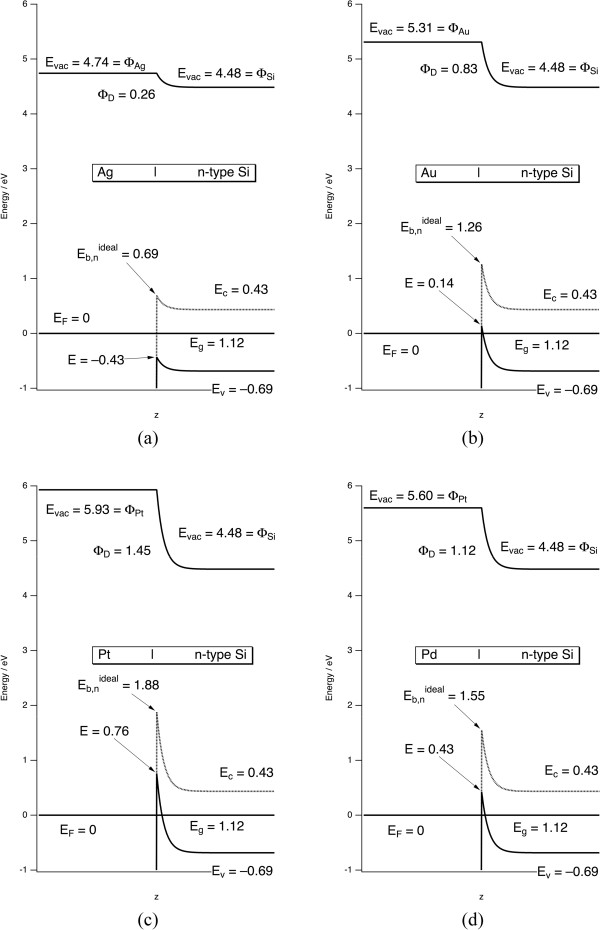
**Band bending at the metal/n-type Si interface for (a) Ag, (b) Au, (c) Pt, and (d) Pd.** All symbols defined as in Figure 1. $E_{b,n}^{\mathrm{ideal}}$ is the Schottky barrier height from Equation 3.

*Φ*_M_ is the metal work function, χ_s_ is the Si electron affinity, and *E*_g_ is the Si bandgap. *E*_vac_(*z*) is the vacuum energy in Si as a function of the distance from the interface *z. E*_vac, Si bulk_ is the constant value of *E*_vac_ deep in the Si bulk. *Φ*_*D*_(*z*) is the value of band bending, which ranges from zero in the bulk to a maximum of *Φ*_*D*_ at the interface. The precise shape and width of the space charge layer are not important, which for convenience is approximated by a simple exponential function to smoothly connect the limiting values at the interface and in the bulk. The Fermi energy is used as the origin, *E*_F_ = 0. The values of these parameters, the standard electrochemical potentials *E°*, and the calculation results are summarized in Table 1. The resulting band diagrams are shown in Figures 1 and 2.In textbooks, it is commonly shown that bands bend upward in n-type Si and downward in p-type Si. Furthermore, it is common to observe upward band bending for n-type Si and downward band bending for p-type Si in aqueous solutions. However, the Schottky-Mott relationships show that upward or downward band bending of the metal/Si interface is controlled by whether the work function of the metal or that of Si is greater. As it turns out, the work functions of three very commonly encountered metals - namely, those of Al, Cu, and Ag - are all lower than the work function of p-type Si but greater than n-type Si. Therefore, the interfaces of Al, Cu, and Ag with Si all conform to the commonly expected trends. Al and Cu are of lower utility in metal-assisted etching. Therefore, the results of calculations only for Ag/Si are shown in Figures 1a and 2a.

Three other commonly used metals for metal-assisted etching, all of which can be deposited by galvanic displacement deposition from solution, are Au, Pt, and Pd. These are all high work function metals compared to Si. In all three cases, the bands bend upward.

As discussed by Tung [14], the Schottky-Mott relationships are an approximation to the true Schottky barrier height because the presence of surface states, reconstructions, or lack of an abrupt interface can lead to lower values. This is corroborated by comparison of the experimental values on n-type Si to the calculated values in Table 1. The values for Ag are close to the ideal value. In all other cases, interfacial chemical and structural changes reduce the barriers below the ideal values. However, the shape of the band bending is always correctly predicted by the Schottky-Mott relations. Therefore, they can be used to characterize the qualitative shape of the bands at the interface, and deviations from ideal character will not be important for hole injection into the valence band as discussed below.

*It is not the Schottky barrier itself that is of interest; rather, it is band bending and the energy of the Si valance band at the interface that are important.* This is because a hole must be transferred from the metal to the Si valence band to induce etching. The Schottky-Mott analysis allows us to calculate the energy of the Si valence band maximum at the interface, which is labeled *E* in Figures 1 and 2. Holes naturally relax to the highest available energy in a band, whereas electrons relax to the lowest energy in the band. The definition of the Schottky barrier height is the energy required to move a charge carrier from the metal to the Si interface; however, the carrier changes from p-type to n-type Si. On p-type material, the Schottky barrier height is the energy required to move a hole from the metal to the Si valence band at the interface. Therefore, the Schottky barrier height is the same as the energy of the Si valence band maximum at the interface. On n-type material, the Schottky barrier height is the energy required to move a hole from the Si conduction band at the interface to the metal. This value is not directly relevant to the discussion of etching. Rather, it is again the energy of the Si valence band maximum at the interface *E* that is required. A nonideal interface may introduce gap states between the conduction and valence bands, which affects the Schottky barrier height. However, the introduction of gap states does not change *E*. Therefore, any inaccuracies in the Schottky-Mott relationships will not change the direction of band bending and should not affect the conclusions of the model presented here.

Figures 1 and 2 show that Ag is clearly different than all other metals. Holes injected into Ag are more stable in Ag than they are either in Si or at the Ag/Si interface. In the three other cases, holes injected into the metal should immediately move to the metal/Si interface where band bending will hold them. *Therefore there should not be any diffusion of holes away from the metal particles in any case and Ag cannot inject holes into Si.* Nonetheless, metal induced etching is observed for all four of these metals and etching is observed away from this interface as evidenced by photoluminescent por-Si formation surrounding the metal nanoparticle. These observations call for an alternative mechanism to explain etching.

I propose that rather than thinking of the metal particles as sources of holes, they should be thought of in terms of charged particles with some density of holes injected by the oxidizing agent. The charge they hold creates an electric field in their vicinity. The potential difference induced by this electric field will change the hole density in the region around the nanoparticles including regions far from the nanoparticle just as would the application of a bias at a nanoelectrode. With a sufficiently large field, the hole density can be raised in the surrounding area sufficiently to facilitate electrochemical etching or even electropolishing, just as in anodic etching when the entire sample rather than just a local portion of the sample is biased.

Using the methods we previously developed [4] to determine the stoichiometry in stain etching without a metal catalyst, we have found that the stoichiometry of both hole injection and H_2_ production vary for the four different metals shown here. We have shown that stain etching was dominated by a valence 2 process [4]. The observation of strong visible photoluminescence was confirmation of the production of nanocrystalline nanoporous Si. Metal-assisted etching using VO_2_^+^ as the oxidant in the presence of a few percent of a monolayer of Ag or Au nanoparticles exhibited the same stoichiometry. In the presence of Pt, a valence 4 process dominated, which led to rapid production of photoluminescent nanoporous Si. Pd acted much differently. Whereas none of the other metals induced etching in the absence of VO_2_^+^, consistent with prior reports [22], we found that etching at a very slow rate begins in the presence of Pd even in the absence of VO_2_^+^. In addition, whereas the rate follows steady first-order kinetics with respect to VO_2_^+^ consumption, just like all the other metals and stain etching in the absence of metals, neither H_2_ production nor the valence of etching is constant for Pd. Etching in the presence of Pd is at first dominated by electropolishing and then proceeded by a mixture of electropolishing and valence 2 porous Si production. In all four cases, the rate of etching in the presence of a metal is significantly faster than for stain etching, i.e., the metal nanoparticles catalyze the injection of holes compared to the rate at a bare Si surface. Preliminary results [23] on the stoichiometry of metal-assisted etching have been reported, with a detailed report to follow (Kolasinski KW, Barclay WB, Sun Y, Aindow M: The stoichiometry of metal assisted etching of Si in V_2_O_5_ + HF and HOOH + HF solutions. In preparation).

### The mechanism of metal-assisted etching

We need to explain the production of an etch track that is very close to the size of the metal particle and the formation of porous Si remote from the particle. From the results of anodic etching [6,24,25], it is well known that there are three electrochemical pathways for Si etching: (1) current doubling (valence 2 process), which leads to the formation of visibly photoluminescent nanoporous Si, (2) current quadrupling (valence 4 process), which leads to visibly photoluminescent nanoporous Si, and (3) electrochemical oxide formation (valence 4 process) followed by chemical removal of the oxide by HF(aq), which leads to electropolishing. Electropolishing occurs above a critical voltage/current density, which can be related to a nonlinearity introduced by water dissociation, which is a precursor to oxide formation [6]. When concentrations and voltages are appropriately adjusted, etching on the edge of the electropolishing regime can lead to current oscillations caused by competition between oxide formation and the various etching processes [26-28].

Our results indicate that stain etching [4] as well as etching in the presence of Ag and Au [23] are dominated by the current doubling pathway. Etching in the presence of Pt is dominated by the current quadrupling pathway. In contrast, the initial lack of nanoporous Si in the presence of Pd indicates that etching is dominated by electropolishing, though it is subsequently accompanied by current doubling etching.

**Figure 3:**
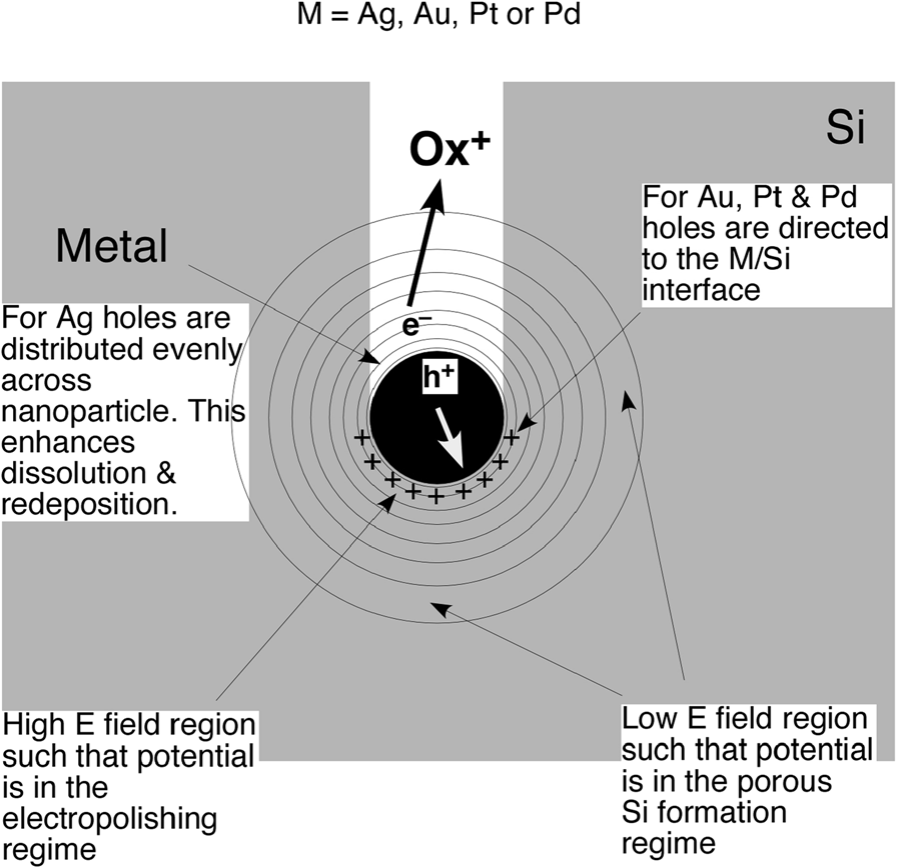
**The mechanism of metal-assisted etching.** Charge accumulation on the metal nanoparticle generates an electric field. Close to the particle, the effective applied voltage is sufficient to push etching into the electropolishing regime, facilitating the formation of an etch track approximately the size of the nanoparticle. Further way, the lower voltage corresponds to the porous silicon formation regime.

How does the metal nanoparticle catalyze electropolishing localized to the nanoparticle/Si interface but also the formation of nanocrystalline por-Si remote from the nanoparticles? The proposed mechanism is illustrated in Figure 3. Rather than injecting holes directly into Si, the positive charge trapped on the metal nanoparticle or at its interface with Si creates an electric field, which turns the nanoparticle into a local anodic power supply. If the voltage is high (above approximately 2 V), anodic etching will enter the electropolishing regime [29]. This would explain the formation of an etch track roughly the size of the metal nanoparticle. Simply estimating the electrical potential *V* induced by a charge *q* at a distance *r* from the center the metal nanoparticle with *V*(*r*) = (4*πϵ*_0_)^- 1^(*q*/*r*), it is found that injection of seven holes into a 5-nm radius nanoparticle will lead to a voltage that exceeds 2 V at the nanoparticle/Si interface. For n-type Si, avalanche breakdown induced etching in the dark is observed for a bias in excess of 10 V [29]. Injection of 35 holes would be sufficient to induce a 10-V bias at the nanoparticle/Si interface. Therefore, it is suggested here that a steady state charge imbalance, in which an excess of holes builds up on the metal nanoparticle due to electron transfer to the oxidant in solution, causes the nanoparticle to act as a localized power supply. For a sufficiently large charge imbalance, the electric field generated by the nanoparticle will be able to engender anodic etching not only at the nanoparticle/Si interface but also deeper into the surrounding Si.Electropolishing will occur at the nanoparticle/Si interface where the potential is highest. Farther away from the metal/Si interface, the electric field is high enough to induce either valence 2 or valence 4 etching and the production of nanocrystalline porous Si. A porous layer surrounding the metal/Si interface would allow for transport of the etchant solution to the interface, which will facilitate etching and the transport of both reactants to and products away from the reactive interface. The oxidant primarily injects holes at the top of the metal nanoparticle rather than at the metal/Si interface, as illustrated in Figure 3.

## Conclusions

The band structure of the metal/Si interface does not facilitate the diffusion of charge away from a metal after an oxidant has injected a hole into the metal. Therefore, the holes injected into the metal are not directly available to induce etching in Si. It is proposed here that the catalytic injection of holes by an oxidant in solution to a metal (film or nanoparticle) in metal-assisted etching (MAE) leads to a steady state charge imbalance in the metal. This excess charge induces an electric field in the vicinity of the metal and biases the surrounding Si. Close to the metal, the potential is raised sufficiently to induce etching with electropolishing character. Further away from the metal, the potential is sufficient to induce etching that leads to the formation of porous silicon by either a valence 2 or valence 4 process. The balance between valence 2 etching, valence 4 etching, and electropolishing varies depending on the chemical identity of the metal.

## Competing interests

The author has no competing interests to declare.

## Authors' contribution

KWK performed all calculations, produced the figures and drafted the manuscript before approving the final manuscript.

## Authors' information

KWK is a Professor of Chemistry as well as a Chartered Chemist (Royal Society of Chemistry) with a Ph.D. in Chemical Physics from Stanford University and a B.S. in Chemistry from the University of Pittsburgh.
